# Salivary beta amyloid protein levels are detectable and differentiate patients with Alzheimer’s disease dementia from normal controls: preliminary findings

**DOI:** 10.1186/s12883-018-1160-y

**Published:** 2018-09-26

**Authors:** Marwan N Sabbagh, Jiong Shi, Moonhee Lee, Lisa Arnold, Yazan Al-Hasan, Jennifer Heim, Patrick McGeer

**Affiliations:** 1 Department of Neurology, Barrow Neurological Institute, St. Joseph’s Hospital and Medical Center, Phoenix, AZ USA; 2 Cleveland Clinic Lou Ruvo Center for Brain Health, 880 W. Bonneville Rd, Las Vegas, Nevada, NV 89106 USA; 3 Aurin Biotech, Vancouver, BC USA

**Keywords:** Alzheimer’s disease, Beta amyloid, Biomarker, Dementia, Saliva

## Abstract

**Background:**

Peripheral diagnostics for Alzheimer’s disease (AD) continue to be developed. Diagnostics capable of detecting AD before the onset of symptoms are particularly desirable, and, given the fact that early detection is imperative for alleviating long-term symptoms of the disease, methods which enable detection in the earliest stages are urgently needed. Saliva testing is non-invasive, and saliva is easy to acquire. A simple, non-invasive saliva test can potentially be used as an adjunct to diagnose AD during its earliest stages.

**Methods:**

Salivary levels of beta amyloid 42 (Aβ_42_) were quantitated with enzyme-linked immunosorbent–type assays. Fifteen AD patients (7 men, mean age 77.8 ± 1.8 years, mean Mini-Mental State Examination [MMSE] score 19.0 ± 1.3) and 7 normal controls (2 men, mean age 60.4 ± 4.7 years, mean MMSE 29.0 ± 0.4) were enrolled.

**Results:**

Salivary Aβ_42_ levels were significantly higher in AD patients than in controls (51.7 ± 1.6 pg/mL for AD and 21.1 ± 0.3 pg/mL for controls, *p* < 0.001). Based on these results, saliva testing appears to be a promising method for detecting AD during its critical early stages.

## Background

The need for a rapid screening method to predict the risk of Alzheimer’s disease (AD) remains an important objective that would inform the decision to initiate early therapy and mitigate the long-term effects and devastating sequelae of advanced AD. Current results of an early objective index have failed to provide a realistic and reasonable screening method. Successful treatment of AD will ultimately depend upon early intervention. In turn, early intervention will depend on detection even before the onset of clinical symptoms. Ideally, biomarker-driven screening could be used for its negative predictive value (i.e., to screen out patients with a low probability of progression).

Peripheral diagnostics for AD continue to be developed and tested. Biomarkers are urgently required for early detection, before clinical signs appear. To date, biomarkers that may detect AD in its early stages involve expensive and invasive procedures. These biomarkers include cerebrospinal fluid analyses of beta amyloid 42 (Aβ_42_), tau, and phosphorylated tau, [1] as well as positron emission tomography scanning for Aβ deposits or tau deposition using mostly fludeoxyglucose (F18) compounds. Although these biomarkers have relatively high specificity and sensitivity, the testing cost is high and access is limited. Recently, the focus has shifted to neurogranin in cerebrospinal fluid.

Biomarkers derived from plasma, serum, and urine has all been explored. Detectable levels of Aβ_42_ in plasma have been reported with variable results. [2, 3] The recent Nature paper demonstrates the utility of assessing plasma Aβ and APP fragments using immunoprecipitation techniques in two separate cohorts from Japan and Australia with very high specificity and sensitivity and correlating the values to PIB PET [4]. Recent studies have reported detection of plasma tau using two different techniques. [5, 6]

A cheap, non-invasive indicator would be an extremely valuable asset for physicians and for patients and their families. A test using saliva is appealing because it is relatively easy to acquire and is non-invasive. Aβ_42_ levels in saliva show promise of being such a biomarker.

Lee et al. [7] demonstrated the feasibility of detecting Aβ_42_ in saliva and other tissues. Bermejo-Pareja et al.[87] analyzed 126 saliva samples from AD cases and controls, as well as 51 saliva samples from Parkinson disease patients. They found that Aβ_42_ levels were significantly elevated in patients with mild to moderate AD but not in patients with severe AD. They found no significant difference between Parkinson disease patients and controls. Mean levels of Aβ_42_ were 2.9–11.70 pg/mL of saliva. Mean proteins averaged 6.6 μg/mL of saliva. They also measured Aβ_40_ levels in saliva. These values were in the range of 21–26 pg/mL of saliva, but no differences were found between AD patients and controls.

We report the detection of salivary Aβ_42_ in prospectively characterized AD patients and controls who were assessed in a memory disorders clinic to demonstrate the feasibility of the use of the protocol and the assay in clinical practice.

## Methods

### Subjects

Fifteen prospectively evaluated patients with mild to moderate AD were enrolled. All 15 met the AD criteria established by the National Institute on Aging and the Alzheimer’s Association (NIA-AA) [9]. Inclusion criteria were Mini-Mental State Examination (MMSE) scores of 10–26 and age ≥ 50 years. For comparison, 8 healthy patients with normal cognitive functioning were included as controls. The controls had no dementia or cognitive impairment and no neurodegenerative disease; they were intact functionally, physically, and socially; were age ≥ 50 years; and had MMSE scores ≥28. We excluded subjects with a medical history of major systemic diseases that could possibly affect cognitive function including other dementias (e.g. DLB, FTD, PPA, PDD, VAD), such as cardiopulmonary failure, hepatic or renal failure, diabetes mellitus, head injury, stroke, or other neurodegenerative disease.

### Approval of human subject protocol

The study was conducted in accordance with the human subject study protocol approved by the Institutional Review Board at Barrow Neurological Institute, St. Joseph’s Hospital and Medical Center, Phoenix, Arizona. The recruitment and informed consent process were conducted by qualified personnel in the Alzheimer’s and Memory Disorders Center at Barrow Neurological Institute. Informed consent was obtained from each participant.

### Specimen collection

Saliva specimens were collected from all 15 patients and all 8 controls. Participants were asked to spit directly into a collection tube labeled with a unique identifier for each participant. The saliva was collected passively (passive drool) without any stimulant or stimulation. Immediately after collection, specimens were stored at room temperature until shipment, as described below.

Specimens were batch shipped via ambient shipment to Aurin Biotech, Inc., and the University of British Columbia under a signed materials transfer agreement. Upon receipt at Aurin or the University, specimens were analyzed immeditely. Residual specimens may be stored at 4 degrees Celsius for up to 1 year after collection..

### Laboratory analysis

Saliva specimens were stored at room temperature until analyzed. The protocol described by Lee et al. [7] was used. Briefly, the Aβ_42_ level in the saliva samples was determined by first adding 2–3 mL of saliva to tubes containing 0.5 mg thioflavine S (Sigma-Aldrich Corp., St. Louis, MO), to prevent Aβ_42_ aggregation. Then 0.5 mg sodium azide (Thermo Fisher Scientific, Inc., Suwanee, GA) was added to prevent bacterial growth. In typical assays, amounts of the saliva mixture varying from 0 (control) to 300 μL were added to the microwells, and enzyme-linked immunosorbent assay (ELISA)–type assays (Aurin Biotech, Inc.) were carried out as described by Lee et al. [7]. Each sample was analyzed in triplicate.

### Statistical analysis

We used the t-test for comparing differences in mean values for the two groups. Data are reported as means and SDs. Significance was defined as *p* < 0.05. An ANCOVA analysis was conducted with Abeta as the dependent variable and age as the covariate..

## Results

The mean age of the 15 AD patients (7 men and 8 women) was 77.8 ± 1.8 years, and the mean age of the 7 controls (2 men, 5 women) was 60.4 ± 4.7 years. The mean MMSE scores for the patients and controls were 19.0 ± 1.3 and 29.0 ± 0.4, respectively. The AD patients were significantly older (t_20_ = 4.25, *p* < 0.05) and more impaired per their MMSE scores (t_19_ = 5.53, p < 0.05) than controls.

**Fig. 1 a:**
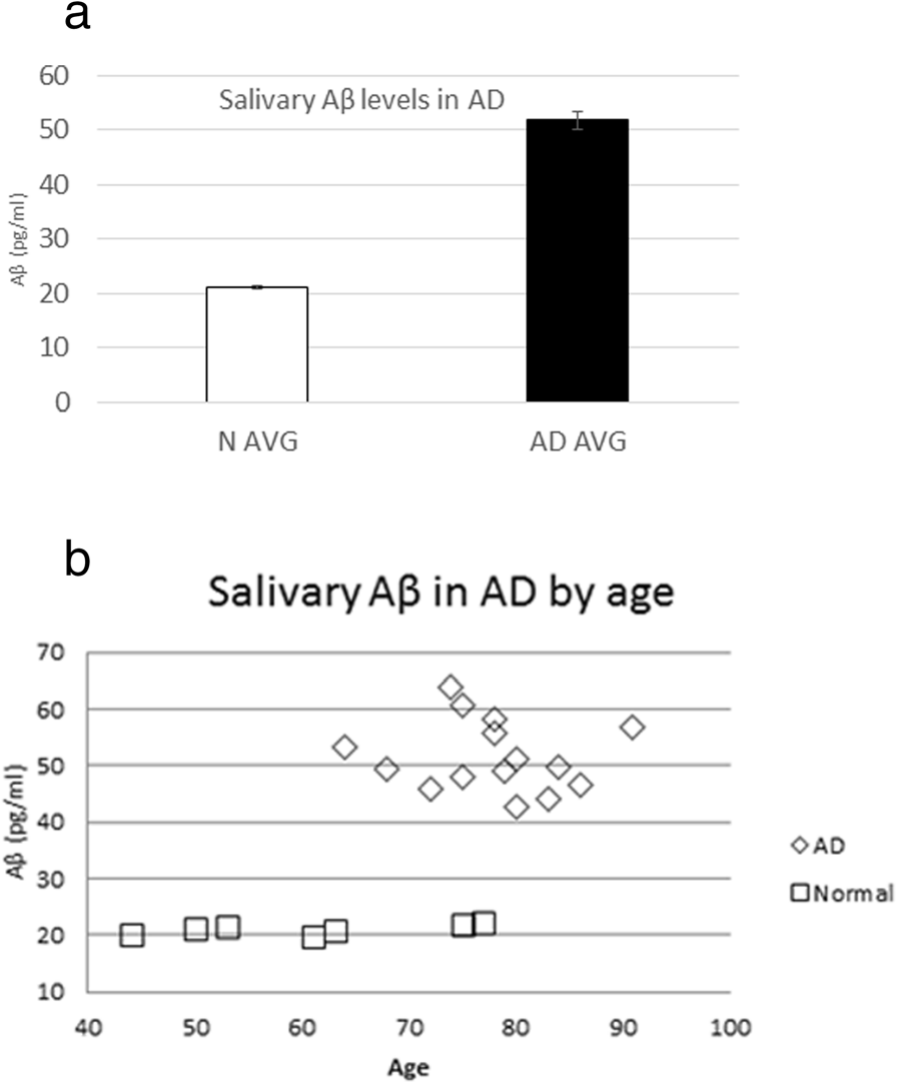
Salivary Aβ_42_ levels (pg/ml) were significantly higher in patients with Alzheimer’s disease than in normal controls. **b** Salivary Aβ_42_ levels (pg/ml) depicted by age. The values are not age dependent (F = 2.27, p-0.14)

After saliva levels were stabilized and mixed with an anti-bacterial agent, we quantitated the Aβ_42_ in a series of samples using ELISA-type assays (Aurin Biotech, Inc.). The Aβ_42_ levels in saliva were found to be significantly higher in AD patients than in controls (51.7 ± 1.6 pg/mL for AD patients and 21.1 ± 0.3 pg/mL for controls, *p* < 0.05) (Fig.1a). The intra assay coefficient of variation (CV) was 3.10 for AD and 1.34 for controls. When the ANCOVA was performed controlling for age, age was not significant (F_1,18_ = 0.02, *p* = 0.884). (Fig.1b).

## Discussion

The results of this study demonstrate and reinforce the feasibility of prospectively collecting and analyzing saliva for the detection and quantification of salivary Aβ_42_. Our analysis of the quantification of Aβ_42_ in the saliva of patients with mild to moderate AD compared to Aβ_42_ in the saliva of controls demonstrated that AD patients had a 2.45-fold increase in Aβ_42_. Thus, the saliva levels of Aβ_42_ were significantly higher (t_20_ = 12.8, *p* = 2.8 × 10^− 4^) in AD patients than in controls. This adds to the previous reports [6] in several ways. First, it demonstrates the feasibility of salivary specimen collection for the detection of Aβ_42_. Second, it shows that the assay is reproducible in that the cohort used in this report does not overlap at all with the first report. Third, the current cohort uses a prospectively characterized clinical sample.

The use of salivary Aβ_42_ as a biomarker for AD is highly innovative compared to more traditional detection methods (cerebrospinal fluid, imaging, and blood studies). The results of this simple, non-invasive test indicate its potential use as an adjunct for the diagnosis of AD or as a screening measure for its negative predictive value to exclude patients with a low probability of disease progression.

The test described measures salivary levels of Aβ terminating at position 42 (Aβ_42_). Previous studies have found Aβ_42_ to be produced in other organs [Lee et al. 2017 7], thus establishing the generality of its production. Brain deposits of this peptide are characteristic of AD. Biomarker studies indicate that these brain deposits commence a decade or more before the clinical onset of AD. Similar to the results of other studies, [7, 8] our findings indicate that Aβ_42_ is elevated in the saliva of patients with AD. Thus, our data support elevated salivary Aβ_42_ as a potential biomarker for AD. The current study replicates the findings of Lee et al. [7] is the amount of amyloid detected is > 2× higher in AD compared to NC. These differences are larger than the differences reported by Bermejo-Pareja [8] possibly because the assay method differs somewhat.

Our report contrasts with previous studies. Shi et al. [10] used high sensitivity mass spectrometry to detect salivary Aβ and tau. They found that salivary tau was elevated but Aβ_42_ was not detectable. How can these findings be explained? Mass spectrometry is able to detect the molecular weights of the proteins. Since there are multiple forms of Aβ, the mass spec might not be detecting the species of Aβ that we are detecting in our study. The main difference in accounting for the detectable Aβ in our study is that we used an ELISA assay with antibodies specifically able to bind to Aβ_42_.

What these results do not indicate is the source of the Aβ. Is Aβ_42_ endogenous to the salivary gland? Future studies might want to explore the histology of salivary glands to determine if salivary glands produce Ab forms similar to the CNS. Amyloid is produced in detectable amounts in a variety of organs [7]. Identifying the source will be important in determining the clinical utility.

In this study, we find that age did not affect the findings even when controlled for. The group of controls in our study was significantly younger than the group of AD patients (60.4 ± 4.7 years vs. 77.8 ± 1.8 years, *p* < 0.05). In contrast, in the Lee et al. [7] study, almost identical levels of salivary Aβ_42_ were found in AD patients and controls aged 15 to 92 years.

Does salivary Aβ_42_ reflect the level of Aβ in the central nervous system? Does salivary Aβ_42_ come from another source? Future studies will explore the source. Future studies will involve collecting saliva specimens across all adult age groups to analyze, compare, and determine the stability of Aβ_42_ by age. Future studies will also assess the specificity and sensitivity of the salivary test in patients with mild cognitive impairment, Lewy body disease, primary progressive aphasia, and Parkinson disease. Other studies to be completed include test-retest validity, and multi-lab assay validity for standardization.

Ultimately, the results of the salivary test for an individual will be correlated with the amyloid status of that person. Future studies will address a variety of questions: Is amyloid positivity associated with higher levels of Aβ_42_? Can the assay method be adapted to detect and quantify other biomarkers, such as tumor necrosis factor-α, interleukin-6, and interleukin-1β? Do salivary levels of Aβ_42_ change with or correlate with the severity of dementia? Even more development is required, including test-retest validity, multi-laboratory validation, and identification of confounders of diurnal variations. Given the strength of the results from in this study, salivary Aβ_42_ warrants further investigation as a potential biomarker for mild to moderate AD.

## Abbreviations

AD: Alzheimer’s disease
Aβ: beta amyloid
ELISA: enzyme-linked immunosorbent assay
MMSE: Mini-Mental State Examination
NIA-AA: National Institute on Aging and Alzheimer’s Association
